# Wettability of Metal Surfaces Affected by Paint Layer Covering

**DOI:** 10.3390/ma15051830

**Published:** 2022-02-28

**Authors:** Stanislaw Pogorzelski, Katarzyna Boniewicz-Szmyt, Maciej Grzegorczyk, Pawel Rochowski

**Affiliations:** 1 Institute of Experimental Physics, Faculty of Mathematics, Physics and Informatics, University of Gdańsk, Wita Stwosza 57, 80-308 Gdansk, Poland; stanislaw.pogorzelski@ug.edu.pl (S.P.); maciej.grzegorczyk@ug.edu.pl (M.G.); 2 Department of Physics, Gdynia Maritime University, Morska 81-87, 81-225 Gdynia, Poland; k.boniewicz@wm.umg.edu.pl; 3 MGE, Lipowa 7, 82-103 Stegna, Poland

**Keywords:** metallic substrata, contact angle, wettability models, contact angle hysteresis, surface wettability energetics, paint layer effect

## Abstract

The aim of the work was to quantify the surface wettability of metallic (Fe, Al, Cu, brass) surfaces covered with sprayed paints. Wettability was determined using the contact angle hysteresis approach, where dynamic contact angles (advancing Θ_A_ and receding Θ_R_) were identified with the inclined plate method. The equilibrium, Θ_Y_, contact angle hysteresis, CAH = Θ_A_ − Θ_R_, film pressure, Π, surface free energy, γ_SV_, works of adhesion, W_A_, and spreading, W_S_, were considered. Hydrophobic water/solid interactions were exhibited for the treated surfaces with the dispersive term contribution to γ_SV_ equal to (0.66–0.69). The registered 3D surface roughness profiles allowed the surface roughness and surface heterogeneity effect on wettability to be discussed. The clean metallic surfaces turned out to be of a hydrophilic nature (Θ_Y_ < 90°) with high γ_SV_, heterogeneous, and rough with a large CAH. The surface covering demonstrated the parameters’ evolution, Θ_A_↑, Θ_R_↑, γ_SV_↓, W_A_↓, and W_S_↓, corresponding to the surface hydrophobization and exhibiting base substratum-specific signatures. The dimensionless roughness fluctuation coefficient, η, was linearly correlated to CAH. The CAH methodology based on the three measurable quantities, Θ_A_, Θ_R_, and liquid surface tension, γ_LV_, can be a useful tool in surface-mediated process studies, such as lubrication, liquid coating, and thermoflow.

## 1. Introduction

Surface preparation is an essential step to obtain specific substrates to be applied in several processes, such as corrosive layer deposition, lubrication, spreading rheology, liquid thermoflow control, etc. Solid material wettability is a fundamental property which reveals specific information on the surface chemical structure, component miscibility, and spatial heterogeneity, apart from the surface roughness morphology which is likely to be strongly correlated to the treatment process conditions (relative humidity, temperature, vibrations, process time scale, etc.). The aim of the study was to quantify the surface wettability signatures of metallic (Al, Fe, Cu, brass alloy) surfaces treated with a polymeric paint-coating process. Wetting measurements based on the contact angle (CA) determination are among the most sensitive and commonly realized surface-sensing techniques with an extremely low analysis depth of the order of nm [1]. The determination of solid surface free energy, γ_SV_, and its components is realized by means of several theoretical approaches based on the so-called Young equilibrium contact angle, Θ_Y_ [2]. Recent experimental water wettability studies of metallic surfaces demonstrated that other kinds of metals (Ag, Au, Pd, Pt), apart from the ones studied here, possessed higher γ_SV_ (within the 40–58 mJ m^−2^ range) and lower CA being a of hydrophilic nature [3] in reference to polymers, wax layers, rubber, and gelatinous materials which stand for hydrophobic substrata of low γ_SV_ (18.9–25.7 mJ m^−2^) and high CA values [4]. In a typical experiment, the interfacial drop-surface system is forced to “relax” to the thermodynamically stable state by advancing and receding deformation modes, and then the equilibrium, Θ_Y_, is recovered from dynamic CA measurements, as argued for heterogeneous, composite surfaces in [5]. During the course of this research, the contact angle hysteresis (CAH) routine was used and based on the three measurable quantities: dynamic Cas, advancing Θ_A_, and receding Θ_R_ and probe liquid surface tension, γ_LV_, to determine the solid substratum apparent surface free energy and additional interfacial interaction parameters, as demonstrated in [6]. Apart from the dynamic contact angles, the surface free energy, γ_SV_, 2D adsorptive film pressure Π, works of adhesion, and spreading W_A_ and W_S_ were selected as condition level indicators of the studied metallic clean (reference) and polymeric paint-coated surfaces.

The axisymmetric drop shape analysis profile (ADSA-P) technique was adopted to determine CAs from the sessile drops, although the contact angle hysteresis was evaluated from the sessile drop shape studied with an inclined plate system, as described in [7,8]. The wettability evolution trend was followed with the distribution of experimental points in the CAH versus W_S_ plane to distinguish between two surface processes simultaneously taking place, i.e., surface roughness smoothing and spatial mixing of the paint mixture components [9]. Confocal microscopy studies allowed the 3D surface architecture evaluations by means of the advanced image analysis programs [10] and to find correlations between the surface wettability parameters (CAH) and roughness fluctuation coefficient, η, and to further test CA-roughness theoretical models of Wenzel and Cassie–Baxter [11,12,13]. The adhesive properties of the surfaces were found to be closely correlated to CAH and Θ_R_ attributed to surface paint components segregation [14]. The effect of relative humidity on CA and γ_SV_ was also addressed [15,16,17,18]. Generally, the paint-treated metallic surfaces revealed more hydrophobic surface properties compared to the untreated surfaces, i.e., Θ_Y_↑, Θ_A_↑, Θ_R_↑, while CAH↓, Π↓, γ_SV_ ↓, W_A_↓, and W_S_↓. The strength of dispersive interactions between water molecules and the substratum was rather strong, i.e., the γ_SV_^d^/γ_SV_ values for the clean surfaces were equal to (0.74–0.77) and decreased to (0.62–0.69) after the surface treatment. The CAH methodology appears to be a sensitive tool for wettability studies of engineering interfacial systems treated in technological processes and can become useful in a long-term, low-cost assessment of surface characteristics under environmental pollution stress.

## 2. Solid Surface Wettability—Background Relations

Wettability of paint layer-coated solid surfaces can be expressed by static, Young CA measurements, whereas the physicochemical processes occurring at the interface and affecting the interaction energetic changes can be quantified in terms of the surface free energy.

The classical surface energy balance equation, known as the Young–Dupre relation (Equation (1)), defines the equilibrium CA in terms of the free interfacial energies of the system [19]:

γ_SV_ − γ_SL_ = γ_LV_ cos θ_Y_ (1)

where γ_SV_, γ_SL_, and γ_LV_ denote the solid/air, solid/water, and water/air interfacial free energies.

The Young equation is applicable only to the ideal solid surfaces, i.e., smooth, flat, chemically homogeneous, non-porous, non-deformable, and insoluble [20]. However, two different dynamic contact angles can be determined on the same solid substratum in contact with the same probe liquid, called the advancing Ѳ_A_ and receding Ѳ_R_ contact angles [21]. The difference between the advancing and receding CAs is defined as the contact angle hysteresis, CAH (=θ_A_ − θ_R_), which can be of the kinetic and thermodynamics types [22]. As shown, the thermodynamic CAH results from the surface roughness or chemical heterogeneity of substrata and represents the surface characteristic property [1]. At least five reasons for CAH appearance can be pointed to: surface roughness, chemical microscopic heterogeneity, molecular reorientation, drop size effect, and the liquid penetration into the solid surface. A close correlation was found between the adhesive properties of the surfaces with water-receding CA and CAH, attributed to the liquid surface penetration or surface reconstruction [14].

The effect of surface roughness and its heterogeneity on the apparent contact angle can be expressed by the Wenzel and Cassie relations, respectively. The equation proposed by Wenzel [11]:

cos Ѳ_W_ = *R_f_* cos Ѳ_Y_ (2)

quantifies the effect of the surface roughness. In this approach, the effect of increased surface area (so-called “effective” area) on CA is accounted for. The roughness factor (*R_f_*) introduced is defined as the ratio of effective/projected areas (geometric/nominal areas); Ѳ_W_ stands for the Wenzel angle (contact angle on rough surface actually measured), while Ѳ_Y_ represents the static Young angle evidenced for the corresponding “flat” surface.

The relation proposed by Cassie and Baxter [12]:

cos Ѳ_C_ = F_1_ cos Ѳ_1_ + F_2_ cos Ѳ_2_ (3)

relates the apparent contact angle to the heterogeneity of the surface. Here, Ѳ_C_—the Cassie angle—is the weighted average of the CAs of the two phase-consisting surfaces, F_1_ and F_2_, corresponding to the fractions of the surface occupied by each phase, and Ѳ_1_ and Ѳ_2_ denote CAs of each phase. For very rough surfaces (in the range of CA 90° < θ < 180°), air is likely to be entrapped in capillaries between the liquid and solid substratum. Therefore, the apparent contact angle can be increased as a result of the remaining degree of air on the solid surface. The contact area between liquid and solid is reduced by the entrapped air (here θ_2_ = 180°), and, assuming the Cassie-Baxter approach, one obtains [23]:

cos θ_CB_ = F_LS_ [cos (θ_Y_) + 1] − 1 (3a)

where θ_CB_ is the apparent contact angle for the Cassie-Baxter state, and F_LS_ is the fraction of the liquid–solid interface, where liquid is in contact with a solid surface as opposed to air; hence, 1—F_LS_ is the fraction of the liquid–air interface, and θ_Y_ is Young’s CA at a smooth surface.

In the case of chemically heterogeneous surfaces, Ѳ_A_ corresponds to the low-energy component of the composite surface, and Ѳ_R_ points to the high-energy one [1]. The following relation was formulated for heterogeneous surfaces with a particular patchwork geometry coverage [5]:

cos Ѳ_C_ = ½ cos Ѳ_A_ + ½ cos Ѳ_R_ (3b)

Another equation was proposed where components are mixing approaches at the molecular level:

(1 + cos Θ_C_)^2^ = ½ (1 + cos Ѳ_A_)^2^ + ½ (1 + cos Ѳ_R_)^2^. (3c)

Solid surface free energy determination formalisms are mostly based on Young’s equation employing equilibrium CA data [2]. In contrast, by applying the CAH model developed by Chibowski [6], the relations allowed the solid surface free energy and the related parameters of liquid–solid surface interaction energetics to be evaluated from the three measurable quantities, the surface tension of probe liquid, γ_LV_, and dynamic contact angles, Ѳ_A_ and Ѳ_R_. The surface free energy of a solid, γ_SV_, reads as follows [6]:

γ_SV_ = Π (1 + cos θ_A_)^2^/[(1 + cos θ_R_)^2^ − (1 + cos θ_A_)^2^] (4)

where the interfacial liquid film pressure, Π, is defined as:

Π = γ_LV_ (cos θ_R_ − cos θ_A_). (5)

It is supposed that the surface modified CAH is attributed to the work of spreading *W_S_*. Consequently, the wettability and the strength of adhesion are related by *W_S_*, which can be derived from the work of adhesion, *W_A_*, and the work of cohesion, *W_C_*:

*W_S_* = *W_A_* − *W_C_* (6)

where the components are defined as *W_A_* = γ_LV_ (1 + cos θ_A_) and *W_C_* = 2 γ_LV_ [19] and allows one to characterize the competition between liquid/solid adhesions with a variety of liquids or substrata differing in their polarities [24].

It should be noted that for “non-hysteresis” systems (hardly to be found in nature), where CAH = 0 and Θ_A_ = Θ_R_ = Θ_Y_, Equation (4) can be converted to [21]:

γ_SV_^tot^ = γ_LV_ (1 + cos θ_A_)/2 = *W_A_*/2. (7)

The surface free energy dispersive component, γ_SV_^d^, is given by [21]:

γ_SV_^d^ = γ_LV_ (1 + cos θ_A_)^2^/4. (8)

Dispersion (London) interactions between molecules of water and apolar components appear to be relatively strong and essential. 

## 3. Materials and Methods

The probe liquid surface tension, γ_LV_; with the accuracy of 0.1 mN m^−1^, was controlled with the Wilhelmy plate technique using a filter paper plate connected to the force sensor arm (GM2 + UL5, Scaime, France). The pH measurements of the test liquid were performed with a microcomputer pH-meter (CP-315, Elmetron, Zabrze, Poland) with a universal electrode.

The axisymmetric drop shape analysis profile (ADSA-P) technique has been adopted to determine CAs from the sessile drops (2–3 mm in diameter) [25]. For each surface, 4-8 measurements were performed at different surface locations for the spatial homogeneity evaluation. The equilibrium CAs were measured after 20 s from water drop depositions. The ADSA-P setup was described in detail elsewhere [7]. A CCD monochrome TAYAMA 1/3” B/W CCD camera (Tayama, Tokyo, Japan) and an M501 magnifying microscope (Polypower, Taipei, Taiwan) horizontally oriented were used to acquire sessile drop side images. They were analyzed to derive CAs with ImageJ routine. The obtained CA measurement errors were within ±1°. The contact angle hysteresis was evaluated from the sessile drop shape studied with an inclined plate system, as described in [26]. Briefly, for a drop on a tilted plane (see Figure 1 in [8]), the contact angle of the advancing edge, Θ_A_, increases; simultaneously, the angle of the receding edge, Θ_R_, decreases. At a certain angle of inclination, the retentive force achieves a critical value; later on, the drop starts to move and that gives proper values of dynamic CAs for the studied system. More details on the experimental CAH measurement procedure can be found in [7].

Surface roughness (SR) profiles of the samples were registered with a confocal microscopy system (Axiovert 200M, Carl Zeiss, Jena, Germany), working in the reflection beam configuration [27] and contour GT optical profilometer (Bruker, Ettlingen, Germany). More detailed signatures of 3D sample architecture were derived by means of the image analysis programs (CMEIAS, ImageJ, PHLIP, COMSTAT, Helicon Focus), as demonstrated in [10]. In particular, ImageJ allows us to create image stacks (perpendicularly along vertical *z* axis) leading to 3D projections of the surface morphology. To standardize the surface roughness degree, surface roughness fluctuation dimensionless coefficient, η, was introduced here, defined as: η = SD/*R_rms_*, where *R_rms_* is the root-mean-square value of the vertical roughness distribution, and SD is the standard deviation from the mean.

Four model metal substrata measuring 76 mm × 30 mm × 3 mm were made of Cu, Al, Fe, and brass alloy. All the selected materials are easily available and commonly used in the manufacturing industry: aluminum (AA 7064 purchased from Durallium, JD, Jenho, Chino Hills, CA, USA); stainless steel (T8—a high carbon steel with 0.78–0.84% carbon from Baoshan Iron and Steel Co. Ltd., Shanghai, China); copper (CU-M-02-SAMP from American Elements, Los Angeles, CA, USA); brass alloy (CuZn_36_Pb_3_CW603N from Filto Profiles, Vallirana, Spain). Each test surface was used as received without any additional mechanical surface treatment; the surface roughness of the samples, *R_rms_*, ranged from 2.3 to 5.6 μm. Prior to each test, the substrates were ultrasonically cleaned with acetone and ethanol.

First, CA determination was performed on untreated clean samples; later on, each of the surfaces was sprayed (as recommended by the producer) with paints: colorless (cs) finishing lacquer alkyd resin-based (Baufix, Holz & Bautechnik GMBH, Seelze, Germany); red (r) finishing lacquer alkyd resin-based (Baufix, Holz & Bautechnik GMBH, Seelze, Germany); black (b) multi-purpose-use paint base on polymeric substances (Bondo/Mar-Hyde Corporation, Atlanta, GA, USA); white (w) anticorrosive primer paint (Hirsh-Pol, Osielsko, Poland). Thicknesses of the paint layers, determined with optical microscopy working in a reflection mode, were contained in the range from 8 to 160 μm, depending on the particular substratum and paint applied, as summarized in Table 1. 

**Figure 1:**
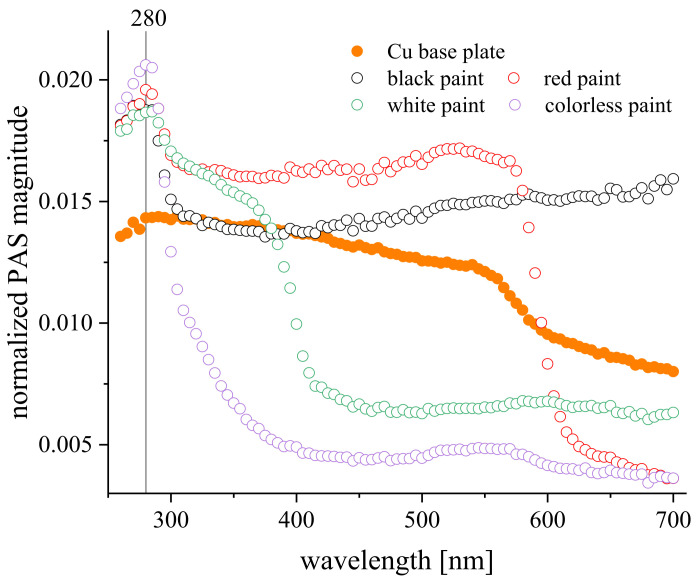
Normalized (with respect to carbon black) photoacoustic spectra for metallic Cu substrata-coated with spread paints; modulation frequency 120 Hz. The spectral band around λ ~ 280 nm points to the presence of a common polymeric component for all the paints investigated, while additional bands show a certain contribution of supplementary pigments.

In order to roughly estimate the chemical similarity between the paints being a mixture of constituents, the photoacoustic spectra (PAS) of the paint-covered metal surfaces were determined with a closed-type photoacoustic cell system, as described elsewhere [28]. Unlike the standard spectroscopic methods, photoacoustic-based modalities allow us to probe spectral characteristics of opaque materials without any need for special sample modifications. The exemplary PAS spectra for the Cu-coated surfaces, shown in Figure 1, revealed the presence of a common polymeric component for all of the paints studied, characterized by an absorption band around λ ~ 280 nm [29], with additional bands corresponding to pigments of particular colors.

As a probe liquid for CA measurements, distilled water with γ_LV_ = 71.7 ± 0.1 mN m^−1^ was taken from a water deionization apparatus (Millipore, conductivity 0.05 S cm^−1^) with pH 5.8 ± 0.1, at ambient temperature, T = 23 °C. Measurements were performed at relative humidity (RH) = 45%.

## 4. Results and Discussion

### 4.1. Equlibrium Contact Angle-System Themodynamic Stability

It should be pointed out that most solid surface energy quantification approaches based on Young’s equation employ the equilibrium CA, which cannot be obtained on practical surface-modified substrata, as addressed in [1]. The sessile drop technique-originating, so-called static CAs, are considered here. The above Equations (1) and (6) and for *W_A_*, i.e., the thermodynamic work of wetting, are valid for the system where phases are in the mutual equilibrium. In fact, the solid surface is said be in equilibrium with the saturated vapor pressure, *p*_0_, of the liquid at measurement temperature (at 100% relative humidity, RH). In addition, Decker and Garoff argued that CA can relax to a lower energy state (of lower CA value) if the applied external energy is high enough to partially force the three-phase contact line to overcome the energy barrier of the metastable state [30]. In order to do that, some novel experimental techniques for the “stable equilibrium” or “ideal equilibrium” CA determination based on the vibration of the system were developed [31]. The problem of wetting transitions on rough surfaces resulting from external stimuli was addressed in [32]. In this experiment, the interfacial drop-surface system was forced to “relax” to the thermodynamically stable state by the advancing and receding deformation modes. The static equilibrium, Ѳ_Y_, contact angles measured directly and derived from CAH data, Ѳ_C_, i.e., from Equations (3b) and (3c), with the corresponding γ_SV_ derived from γ_SV_^a–c^ = ½ *W_A_* = ½ γ_LV_ (1 + cos θeq), are collected in Table 2.

As found for each of the studied surfaces, Ѳ_Y_ > Ѳ_C_^b^ ~ Ѳ_C_^c^. The static CA difference ranges from 3.0 to 12.1°, which leads to lower γ_SV_ by 9–13 mJ m^−2^, for the direct measurement in reference to the CAH-derived ones. Ѳ_C_^b^ and Ѳ_C_^c^ values appeared to be in agreement with the reference data reported for metallic surfaces by others [33,34,35,36]. Since Ѳ_C_^b^ ~ Ѳ_C_^c^ are very close to each other (within 2–3°), the kind of mixing model of components in the composite surface is of secondary importance in determining Ѳ_C_. As a result, the dynamic contact angle hysteresis procedure can lead to realistic, equilibrium CAs corresponding to the thermodynamically stable state of the interfacial system. The studied surfaces were all of hydrophilic character (0 < Ѳ_Y_ < 90°). The paint-covered surfaces became more hydrophobic in reference to the original ones, where Ѳ_Y_ increased by 12–15°, and γ_SV_ decreased by 5.7–10.5 mJ m^−2^.

### 4.2. Relative Humidity Effect

**Figure 2:**
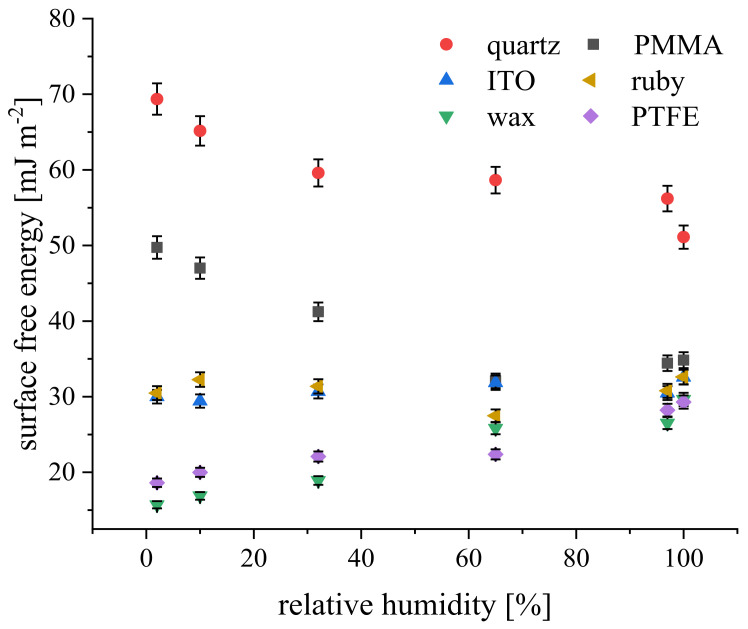
Surface free energy, γ_SV_, versus relative humidity, RH, for solid surfaces of different hydrophobicity.

The effect of humidity on CA and γ_SV_ was addressed in [15,16,17,18,37], for solid substrata of different polarity. Contact angles of aluminosilicate clays appeared slightly affected by RH between 19 and 75%, and a lower CA was detected at 100%, which likely resulted from the expansion of water film adsorption at the clay surface at RH = 100% [17]. Maximum apparent surface free energy of DPPC (dipalmitoylphosphatidylcholine) bilayer on glass was determined at 50% and 90% RH [16]. These changes in the DPPC bilayer wettability were interpreted as caused by water vapor adsorption. Metals and metal oxides are classified as high-energy solids [15], and the monotonic decrease of the total surface energy from around 70 to 47 mJ m^−2^ was reported with an increase of RH for the grid-blasted steel surface. The surface free energy of any clean, high-energy surface exposed to an atmosphere containing water vapor is dependent upon the surface concentration of adsorbed water, likely in a form of micrometer-sized water droplets [37]. Contact angle and the surface energy studies performed on original silicon wafers revealed CA and γ_SV_ dependence on RH only within the range of 10 to 40% [37]. However, for the oxidized silicon wafer CA and γ_SV_, variability depended on RH, changing periodically with an increase of RH [18]. These changes can be attributed to water adsorption on the hydrophilic silicon surface. Our preliminary CA measurements performed on solid substrata enclosed in humidity-controlled chambers, where the variable humidity was maintained by using salt solutions, demonstrated a decrease of γ_SV_ with an increase of RH for the solid of hydrophilic character (quartz, poly(methyl metracrylate)—PMMA). The opposite trend was noticed for hydrophobic surfaces (paraffin wax, polytetrafluoroethylene—PTFE), whereas no significant effect was found for indium tin oxide (ITO) alloy and ruby (Al_2_O_3_-based crystal) surfaces, as depicted in Figure 2.

### 4.3. Wettability from Dynamic Contact Angles

The CAH approach allowed for the quantitative evaluations of interfacial interactions by means of wettability parameters, as collected in Table 3, for metallic surfaces free and coated with color spread paints.

As a matter of fact, CAH parameter variability referred to the clean, unaffected surface case rather than their absolute values and represent a useful monitoring tool for the metallic surface modification. 

**Figure 3:**
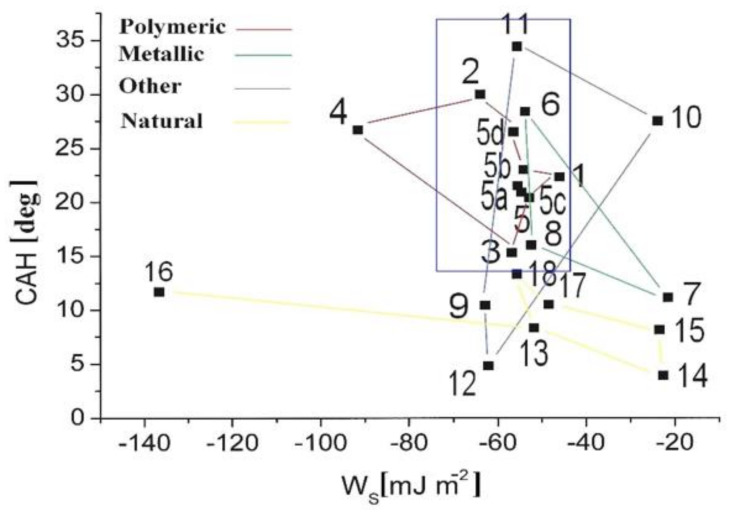
Contact angle hysteresis (CAH) as a function of work of spreading W_S_ for a variety of solid substrata in contact with water; denotations and data from [4] with the blue box area comprising data for the studied paint-covered surfaces.

The metallic surface paint-covering effect revealed the following variability of the CAH parameters: θ_A_↑, θ_R_↑, CAH↓ by a few percent (5–8%); Π exhibited high values (from 46.9 mN m^−1^) and decreased; γ_SV_↑ (for Al and Fe samples) and γ_SV_↓ (for Cu and brass samples) by several percent (11.3–30.0%); W_A_ decreased by 5.6–11.3%, W_S_ became more negative (changing from −46.6 to −68.9 mJ m^−2^). The surface energy parameters exhibited mainly hydrophobic water/solid surface interactions, i.e., the dispersive term, γ_SV_^d^, of the surface free energy was equal to (0.74–0.77) γ_SV_ and decreased to (0.66–0.69) γ_SV_ for the coated surfaces. It can be stated that the strength of the dispersive interactions between the water molecules and model surfaces was rather weak in reference to hydrophobic polymer surfaces (PMMA), with γ_SV_^d^ equal to 0.89–0.90 γ_SV_, whereas for hydrophilic surfaces, the dispersive term contribution was equal to 0.33 γ_SV_ (glass), 0.59 γ_SV_ (steel), and 0.42 γ_SV_ (silicon) [8]. The observed strong CAH on hydrophilic surfaces appeared as a result of the high energy barrier for interfacial liquid density fluctuations [13]. As demonstrated in [38], an increase in CAH leads to the stronger adhesion between the liquid and the substratum. The receding CA is actually the measurement most characteristic of the modified component of the surface, particularly for surfaces modified by adhesive layer deposition creating energy barriers [39]. As θ_R_↑, the relative contribution of most hydrophilic components in the surface free energy increased according to Equation (4). On the other hand, for the same surface, θ_A_↑ is noticed, which points to the selection process of both less hydrophilic and most hydrophilic compounds occupying the outermost solid area in a complex way. There is the further problem of the solid surface free energy calculation, even for a given system with properly determined CA, because of the film pressure, Π—Equation (5) affects the apparent free energy of the solid surface [21]. Noting that the liquid does not spread completely over the solid surface, the so-called film pressure, Π, becomes positive, and the film increases the apparent surface energy. As a result, the Young–Dupre relation should take the modified form [21]: γ_SV_ − γ_SL_ − Π = γ_LV_ cos θ_Y_. The complex surface wettability of the metallic paint-covered surfaces evolution can be reflected in the spatial distribution of the experimental points placed in the 2D space of CAH plotted versus W_S_ [8,9]. Such a plot constructed from the data of comprehensive measurements performed on differentiated model substrata (polymeric, metallic, natural–biological, and composite) obtained in previous authors’ studies ([4]) is presented in Figure 3. On the background data, the blue box covering area CAH (12.9–46.6 mN m^−1^) and *W_S_* (from −73.7 to −45.0 mJ m^−2^) corresponding the studied here surfaces coordinates is also included, for comparison.

**Figure 4:**
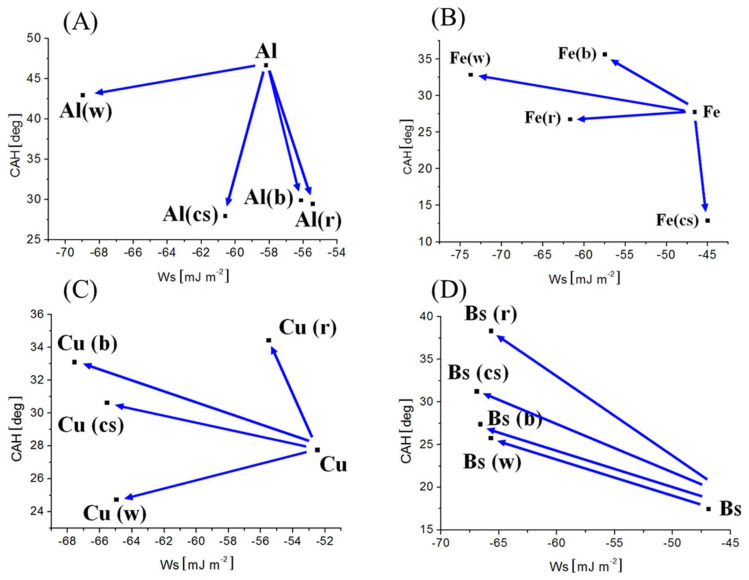
Contact angle hysteresis, CAH, as a function of work of spreading, W_S_, for metallic surfaces: (**A**) Al, (**B**) Fe, (**C**) Cu, and (**D**) brass alloy, clean-reference, and coated with color paints: w—white, b—black, r—red, and cs—colorless (transparent). The data evolution was indicated with the arrows in reference to the clean surface case.

The paint-covered surfaces stand for a complex interfacial system, where several processes, such as roughness smoothing and paint components spatial and temporal segregation take place simultaneously. Moreover, they are base substratum specific, as demonstrated in Figure 4A–D, for each of the surface-modified samples separately.

The experimental point coordinate evolution demonstrated the similarly smooth and homogeneous surfaces for the paint-treated Al surfaces: Al(b), Al(cs), and Al(r) as the reference Al (Figure 4A), since CAH laid in 27–29° (CAH ~ const.), but the component composition is widely distributed (*W_S_* was ranging from −60.6 to −55.4 mJ m^−2^). For Al(w) surface, CAH was close (46.6°) to the reference surface case (42.9°), but the makeup of the components was different as reflected in *W_S_* changing from −58.2 to −68.9 mJ m^−2^, and the increased contribution of more hydrophobic compounds (both θ_R_↑ and θ_A_↑) pointing to the surface hydrophobization process.

The Fe surface treatment, for (w), (b), and (r) paint coverings demonstrated a similar evolution trend of wettability (Figure 4B). These painted surfaces became enriched with more hydrophobic components (both θ_R_↑ and θ_A_↑), and *W_S_* became more negative from −46.6 to −73.7 (w) mJ m^−2^. Fe(cs) surface wettability appeared to be an exception; *W_A_* corresponded almost to the reference one (98.3 and 96.8 mJ m^−2^, respectively) but such a surface was smoother and more homogeneous (CAH drop from 27.7 to 12.8°). 

For paint-covered Cu surfaces, similar component compositions can be noticed for (b), (cs), and (w) colors leading to surface hydrophobization with *W_S_* contained in the range of −67.6 to −65.4 mJ m^−2^ in reference to the clean Cu surface (Figure 4C). However, they represent surfaces of differentiated microroughness and spatial homogeneity reflected in large differences in CAH (from 24.7° (w), 30.6° (cs) to 33.0° (b)). The exceptional wetting signatures revealed the Cu(r)-painted surface, where both the surface roughness and its heterogeneity increase were likely to appear (CAH↑), and hydrophobicity progressed (W_S_ more negative) compared to the reference Cu sample.

The smoothing effect of paints on roughness of the brass surface was evident from the data point distribution in Figure 4D. For all the painted surfaces, paint-forming components were of similar proportions and wetting properties (θ_A_ differed slightly from 85.2–86.2° as well as θ_R_ (54.9–59.5°)), and exhibited comparable adhesive strength, *W_A_* (76.5–77.7 mJ m^−2^), i.e., *W_S_* ~ const. Under such conditions, the smoothing effect was reflected in CAH variability directed parallel to CAH axis. 

### 4.4. Surface Architecture versus Wettability

**Figure 5:**
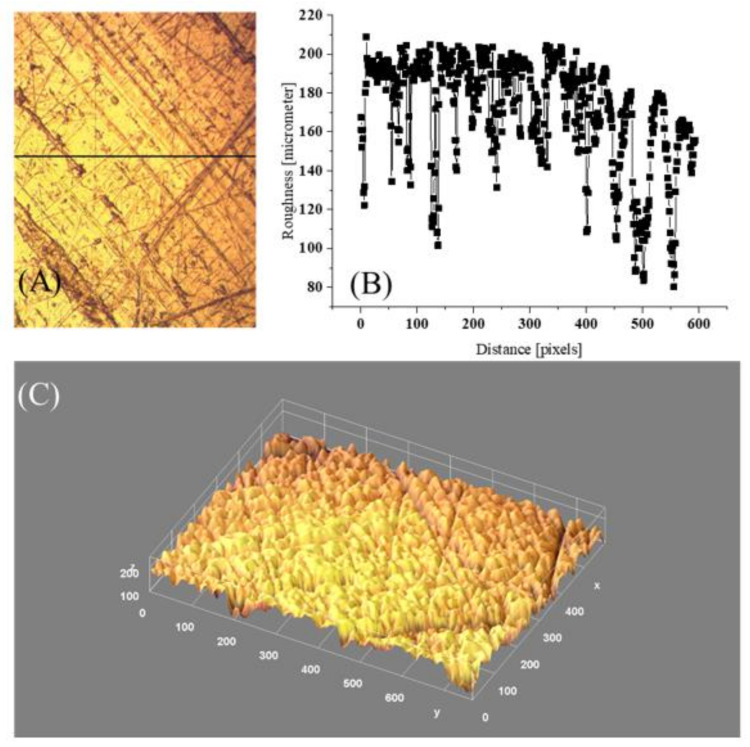
Microscopic images (magnification 400×) of Al (ref) surface architecture (**A**), (**B**) 2D surface profile along the line from (**A**), and (**C**) 3D surface morphology derived with ImageJ procedure; covered area x = 164.4 and y = 209.4 μm, distance scale 36 pixels = 10 μm, roughness fluctuation coefficient η = 0.163.

Surface morphology of the Al (ref) sample is shown in Figure 5A (the surface plot area covers 164.4 × 209.4 μm range); the surface profile along the line from Figure 5A is presented in Figure 5B, and 3D surface architecture is demonstrated in Figure 5C. The *R_f_* parameter = 2.71, and *R_rms_* = 3.5 ± 0.2 μm. As argued in [21], the apparent surface free energy decreases gradually with the increase of the roughness ratio, *R_f_*. Moreover, for a very rough Al surface for which the undulation profile is shown in Figure 5B, the surface free energy evaluated from the water CA using the Wenzel approach (Equation (2)) is as low as 18 mJ m^−2^. Consequently, for large enough roughness (i.e., enough large ratios *R_f_* = A/A_0_), the Cassie–Baxter state appears to be the thermodynamically stable state for the interfacial system [13]. The F_LS_ fraction can be determined from Equation (3a): F_LS_ = (cos Θ_CB_ + 1)/(cos Θ_Y_ + 1). Since from the measurement, Θ_CB_ = 56.6° and Θ_Y_ = 49.3° (for flat polished surface), F_LS_ = 0.94, and the surface fraction is covered with the entrapped air under the liquid phase = 1 − F_LS_ = 0.06. 

By applying the simplified model (see Figure 6 in [13]), a pressure which has to applied to a cylindrical cavity to the droplet covering the cavity inlet area can be evaluated. The liquid tends to bend inwards the cavity if the pressure exceeds the critical Laplace capillary pressure p_c_ = −2 γ_LV_ cos (Θ) R^−1^, where R is the radius of the cavity. In such a case, the liquid will be squeezed into the capillary. By analyzing the surface profile from Figure 5B, it was found that the mean capillary-like scratches had a cavity radius distribution with the mean R = (4.0 ± 2.5 μm). The required pressure is p_c_ = 19,734 Pa (i.e., = 0.19 p_a_, where: p_a_ is the normal ambient atmospheric pressure). In the framework of the presented model, if Θ < 90° (hydrophilic surface), p_c_ < 0, and the liquid is supposed to fill out the cavity (as assumed in Wenzel state). On the other hand, for Θ > 90° (hydrophobic surface), p_c_ > 0, and very high pressures must be applied to squeeze liquid into capillaries.

The vertical roughness profile, *R_rms_*, better characterizes the rough surface wettability than the R_f_ parameter [21]. It was postulated that the water contact angle (in degrees) is almost linearly dependent on the average, *R_rms_* (μm), for Al modified surfaces (polishing, sandblasting, chemical etching, laser ablation, etc.), and approximated with the function [33]: Θ_Y_ = −14 *R_rms_* + 100. For the Al surface considered here, one obtains Θ_Y_ = 51.0° from the approach in reference to the measured CA value = 56.6°_,_ which reveals a certain agreement with the model-predicted value.

The chemical composition, apart from the surface structure and hydrophobic/hydrophilic properties, affect the wettability [35]. Surfaces of metals and their alloys are coated with oxide films, which results in their hydrophilic properties with high surface energy. The final wettability depends on the history of the surface treatment when the surface processes induct adsorption, chemisorption, or chemical interaction of gases occurring in the ambient environment. Recently, several studies were performed on laser texturing of stainless steel, polymeric surfaces followed by surface modification with organic coating adapted to create composite surfaces with different surface roughness (*R_f_*), morphologies, and wettability [40,41,42]. It is known that high energy surfaces (of hydrophilic character) are enriched in polar functional groups such as, –CO, –NH_2_, –OH, –COOH, whereas the hydrophobic surfaces contain non-polar groups (alkyl, fluoroalkyl, –SH, etc.). To conclude, assuming that the surface morphologies of all the surfaces are similar, the surface hydrophilicity should increase with an increase of oxygen content, and increased carbon content should lead to hydrophobic properties.

**Figure 6:**
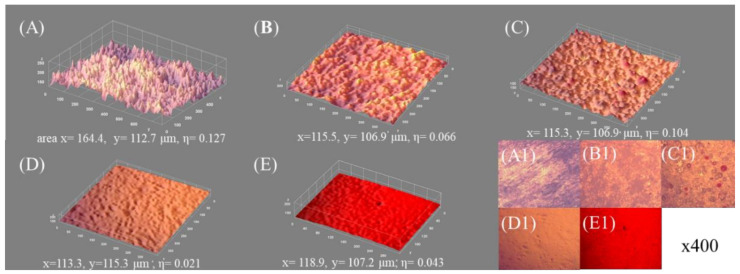
3D surface morphology and microscopic images (magnification 400×) of brass (clean) (**A** and **A1**, respectively) and covered with paints of different kinds: (**B**,**B1**) colorless, (**C**,**C1**) white, (**D**,**D1**) black, (**E**,**E1**) red; distance scale: 36 pixels = 10 μm; image-covered surface areas and surface roughness fluctuation coefficients, η, are given underneath.

Microscopic registrations and 3D-studied surface micro architecture, for brass (free) and its paint-coated surfaces are depicted in Figure 6A–E. Generally, the metal surface paint-treatment was manifested by lowered *R_rms_* and η values, the flattening of valleys, and closing of inter-grain regions, slits, and cracks.

CAH was found to be greater in the case of rough surfaces, but it is mainly attributed to chemical interactions and heterogeneities [36]. The relationship for CAH as a function of roughness, *R_f_*, was already postulated [43]. For a homogeneous interface a roughness increase (high *R_f_*) results in an increase of CA hysteresis (high CAH values). For a composite surface with regions of different wettability, it leads to both high CA and low CA hysteresis according to the relation: CAH = *R_f_* [(cos Ѳ_R0_ − cos Ѳ_A0_)/(*R_f_* cos Ѳ_eq_ + 1)^1/2^], where Ѳ_R0_, Ѳ_A0_, and Ѳ_eq_ (~ Ѳ_Y_) are the receding, advancing, and equilibrium contact angles, respectively, derived for a smooth interface. 

**Figure 7:**
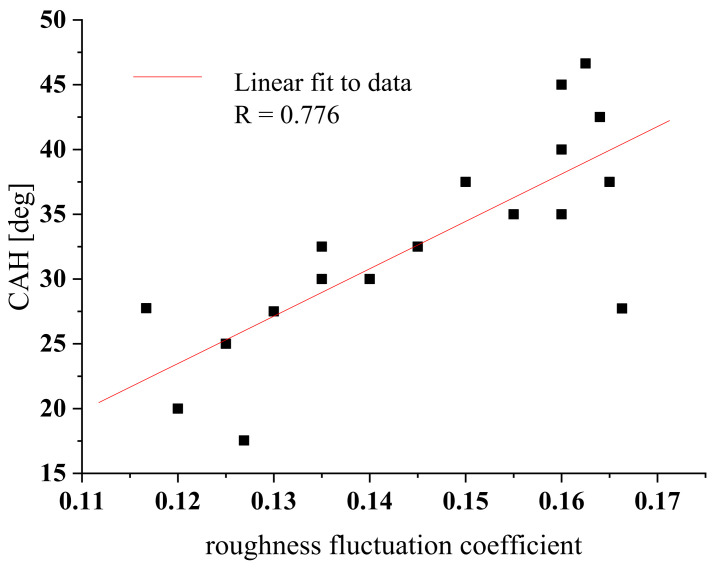
Contact angle hysteresis, CAH, versus surface roughness fluctuation coefficient, η, for metallic clean and paint-coated substrata. The ±2.0° CAH error bars were omitted to enhance the data presentation.

Values of η demonstrated the following trend: η(ref) > η(w) > η(cs) > η(r) > η(b), as given in each picture from Figure 6A–E. Generally, the linear relation between CAH and the roughness coefficient, η, was formulated in the form: CAH = A + Bη, where A = −20.45 ± 10.85, and B = 366.08 ± 74.17; correlation coefficient R = 0.78, valid for all the studied surfaces, as depicted in Figure 7.

## 5. Conclusions

The paint-coated treatment of originally high surface energy, hydrophilic metallic substrata changed the interfacial force balance due to different interactions, and the γ_SV_^d^/γ_SV_ ratio originally being equal to (0.74–0.77) γ_SV_ decreased to (0.62–0.69) γ_SV_. A set of the surface wettability energetics parameters revealed the general trend of their variability as follows: Θ_Y_↑, Θ_A_↑, Θ_R_↑_,_ CAH↑, γ_SV_↓, *W_A_*↓, and *W_S_* less negative, characteristic for the surface hydrophobization. However, a spatial evolution of the data points distribution in the space of CAH versus *W_S_* made it possible to distinguish between the processes simultaneously taking place, i.e., micro-roughness smoothing, chemical paint components distribution and mixing at the outermost surface; they turned out to be base substratum specific. The surface wettability was mainly attributed to the compositional changes at the interface than to the surface roughness and homogeneity since CAH remained almost unchanged for both the reference and treated samples. An increase in both θ_R_ and θ_A_ for the treated surface confirms such a conceivable explanation. For rough surfaces (roughness parameter *R_f_* >> 1), the stable thermodynamic state of the interface was obeyed by the Cassie–Baxter model rather than by the Wenzel one. The static, equilibrium CA can be derived from the dynamic contact angles approach as proposed here, leading to γ_SV_ values agreeing with the data reported by others for the studied model surfaces. High Π values are related to the organized water molecules at the interface mediated by relative humidity, RH. It seems that the surface treatment was affected by the interfacial organization of water molecules in the bulk phase because Π values were apparently higher (29.2–42.9 mN m^−1^) compared to (19.0–28.4 mN m^−1^) for the reference sample.

Large values of CAH were found that are characteristic for hydrophilic surfaces with a high energy barrier. Moreover, CAH appeared to be linearly dependant on the so-called surface roughness fluctuation dimensionless coefficient, which was introduced here as a universal measure applicable to any surface of a certain undulation degree. In this study, we were interested in the surface treatment wettability modifications important from a practical point of view, i.e., for practical applications in lubrication, surface rheology, adhesive layer coating, liquid themoflow, painting, and printing. The presented CAH methodology turned out to an effective tool for the wettability monitoring and quantification of surface-treated solid substrata also exposed to pollution stress pressure seen in natural environment assessments.

## Figures and Tables

**Table 1:** Thickness of paint layers at metallic surfaces [μm]; mean (standard deviation).

Metallic Substratum	Colorless	White	Black	Red
Al	44 (2)	24 (3)	44 (2)	8 (2)
Fe	20 (2)	14 (3)	8 (3)	10 (2)
Brass	40 (3)	8 (2)	104 (6)	16 (3)
Cu	56 (6)	84 (5)	8 (2)	160 (8)

**Table 2:** Static equilibrium contact angles, θ_eq_, and corresponding γ_SV_ for metallic free (ref.) and coated with spread paint surfaces; probe liquid, γ_LV_ = 71.7 mN m^−1^ at 23 °C. The experimental uncertainties and standard deviations are given in brackets.

Substratum	Θ_Y_^a^	γ_SV_^a^	Θ_C_^b^	γ_SV_^b^	Θ_C_^c^	γ_SV_^c^
	[deg]	[mJ m^−2^]	[deg]	[mJ m^−2^]	[deg]	[mJ m^−2^]
Al ref.	67.1 (1)	33.4 (1.5)	58.9 (2.3)	54.3 (1.7)	56.6 (1.8)	55.6 (2.1)
Al (w)	85.4 (1)	28.1 (1.4)	68.2 (2.4)	49.2 (1.6)	65.6 (2.2)	50.6 (2.0)
Al (b)	75.2 (1)	36.7 (1.6)	63.6 (2.3)	51.8 (1.7)	62.4 (2.1)	52.4 (2.0)
Al (r)	74.8 (1)	37.1 (1.6)	63.2 (2.3)	51.9 (1.7)	62.1 (2.1)	52.6 (2.0)
Al (cs)	79.1 (1)	34.7 (1.5)	67.9 (2.4)	49.4 (1.6)	66.8 (2.2)	49.9 (2.0)
Fe ref.	68.0 (1)	42.2 (1.6)	56.8 (2.2)	55.5 (1.8)	55.9 (1.8)	55.9 (2.2)
Fe (w)	83.9 (1)	27.2 (1.4)	75.8 (2.5)	44.6 (1.6)	74.1 (3.1)	45.6 (1.8)
Fe (b)	78.2 (1)	35.1 (1.5)	62.3 (2.3)	52.5 (1.7)	60.7 (2.0)	53.4 (2.1)
Fe (r)	77.6 (1)	34.4 (1.5)	69.2 (2.4)	48.6 (1.6)	68.2 (2.7)	49.2 (1.9)
Fe (cs)	63.7 (1)	45.9 (1.7)	61.9 (2.3)	52.7 (1.7)	61.7 (2.2)	52.8 (2.1)
Cu ref.	70.2 (1)	39.0 (1.6)	61.5 (2.3)	52.9 (1.7)	60.6 (1.9)	53.4 (2.1)
Cu (w)	81.6 (1)	33.0 (1.5)	72.7 (2.5)	46.5 (1.6)	71.7 (2.9)	47.1 (1.8)
Cu (b)	83.9 (1)	30.2 (1.4)	71.0 (2.5)	47.5 (1.6)	69.4 (2.8)	48.4 (1.9)
Cu (r)	70.8 (1)	36.4 (1.5)	62.2 (2.3)	52.5 (1.7)	59.8 (1.9)	53.9 (2.2)
Cu (cs)	79.9 (1)	31.7 (1.4)	70.5 (2.4)	47.8 (1.7)	69.1 (2.8)	48.6 (1.9)
brass ref.	64.0 (1)	43.9 (1.7)	61.4 (2.3)	52.9 (1.7)	61.0 (2.1)	53.2 (2.2)
brass (w)	79.1 (1)	32.5 (1.5)	72.8 (2.5)	46.4 (1.6)	71.8 (3.0)	47.1 (1.8)
brass (b)	77.8 (1)	31.7 (1.4)	72.9 (2.5)	46.5 (1.6)	71.6 (3.0)	47.2 (1.8)
brass (r)	73.4 (1)	30.4 (1.4)	67.4 (2.4)	49.6 (1.6)	65.4 (2.6)	50.7 (2.0)
brass (cs)	72.3 (1)	30.9 (1.4)	71.3 (2.5)	47.3 (1.6)	69.8 (2.8)	48.2 (1.8)

^a^ CA directly measured. ^b^ CA derived from CAH data (Equation (3b)). ^c^ CA derived from CAH data (Equation (3c)). γ_SV_^a–c^ = ½ W_A_ = ½ γ_LV_ (1 + cos θ_eq_) (Equation (7)).

**Table 3:** Wettability parameters derived from dynamic contact angles for metallic surfaces coated with spread paints; water probe liquid, γ_LV_ = 71.7 mN m^−1^, *W_C_* = 143.4 mJ m^−2^ at T = 23 °C. Denotations: ref.—paint free surface; paint color: w—white, b—black, r—red, cs—colorless (transparent). The experimental uncertainties and standard deviations are given in brackets.

Substratum	θ_A_	θ_R_	CAH	π	γ_SV_	*W_A_*	*W_S_*	γ_SV_^d^	γ_SV_^d^/γ_SV_
	[°]	[°]	[°]	[mN m^−1^]	[mJ m^−2^]	[mJ m^−2^]	[mJ m^−2^]	[mJ m^−2^]	[-]
Al ref.	79.2 (1)	32.5 (1)	46.6 (2)	46.9 (1.5)	33.4 (1.5)	85.2 (2.5)	−58.2 (2.7)	25.3 (0.5)	0.76 (0.4)
Al (w)	87.8 (1)	44.9 (1)	42.9 (2)	48.0 (1.5)	28.1 (1.4)	74.4 (2.1)	−68.9 (2.3)	19.3 (0.4)	0.69 (0.3)
Al (b)	77.5 (1)	47.6 (1)	29.9 (2)	32.8 (1.2)	36.7 (1.6)	87.2 (2.6)	−56.1 (2.8)	26.5 (0.5)	0.72 (0.4)
Al (r)	76.9 (1)	47.5 (1)	29.4 (2)	32.2 (1.2)	37.1 (1.6)	87.9 (2.6)	−55.4 (2.8)	26.9 (0.5)	0.73 (0.4)
Al (cs)	81.1 (1)	53.2 (1)	27.9 (2)	31.9 (1.2)	34.7 (1.5)	82.8 (2.4)	−60.6 (2.6)	23.9 (0.5)	0.69 (0.4)
Fe ref.	69.5 (1)	41.8 (1)	27.7 (2)	28.4 (1.1)	42.2 (1.6)	96.8 (2.8)	−46.6 (3.0)	32.7 (0.6)	0.77 (0.5)
Fe (w)	91.7 (1)	58.9 (1)	32.8 (2)	39.1 (1.4)	27.2 (1.4)	69.6 (1.8)	−73.7 (2.0)	16.9 (0.3)	0.62 (0.3)
Fe (b)	78.6 (1)	42.9 (1)	35.6 (2)	38.3 (1.4)	35.1 (1.5)	85.9 (2.6)	−57.5 (2.8)	25.7 (0.5)	0.73 (0.4)
Fe (r)	81.9 (1)	55.2 (1)	26.7 (2)	30.9 (1.2)	34.4 (1.5)	81.7 (2.4)	−61.6 (2.6)	23.3 (0.5)	0.68 (0.4)
Fe (cs)	68.2 (1)	55.3 (1)	12.9 (2)	14.1 (0.8)	45.9 (1.7)	98.3 (2.8)	−45.0 (3.0)	33.7 (0.6)	0.74 (0.4)
Cu ref.	74.5 (1)	46.7 (1)	27.8 (2)	29.9 (1.1)	39.0 (1.6)	90.9 (2.7)	−52.5 (2.9)	28.8 (0.5)	0.74 (0.4)
Cu (w)	84.6 (1)	59.9 (1)	24.7 (2)	29.2 (1.1)	33.0 (1.5)	78.4 (2.3)	−64.9 (2.5)	21.4 (0.4)	0.65 (0.3)
Cu (b)	86.7 (1)	53.6 (1)	33.1 (2)	38.4 (1.4)	30.2 (1.4)	75.8 (2.1)	−67.6 (2.3)	20.0 (0.4)	0.66 (0.3)
Cu (r)	76.9 (1)	42.5 (1)	34.4 (2)	36.6 (1.4)	36.4 (1.5)	87.9 (2.6)	−55.5 (2.8)	26.9 (0.5)	0.74 (0.4)
Cu (cs)	85.1 (1)	54.5 (1)	30.6 (2)	35.5 (1.4)	31.7 (1.4)	77.8 (2.3)	−65.5 (2.5)	21.1 (0.4)	0.67 (0.3)
brass ref.	69.8 (1)	52.3 (1)	17.5 (2)	19.0 (0.9)	43.9 (1.7)	96.5 (2.8)	−46.9 (3.0)	32.5 (0.6)	0.74 (0.4)
brass (w)	85.2 (1)	59.5 (1)	25.8 (2)	30.4 (1.2)	32.5 (1.5)	77.7 (2.3)	−65.7 (2.5)	21.1 (0.4)	0.65 (0.3)
brass (b)	85.9 (1)	58.6 (1)	27.4 (2)	32.3 (1.2)	31.7 (1.4)	76.8 (2.3)	−66.6 (2.5)	20.6 (0.4)	0.65 (0.3)
brass (r)	85.2 (1)	46.9 (1)	38.3 (2)	42.9 (1.4)	30.4 (1.4)	77.7 (2.3)	−65.7 (2.5)	21.1 (0.4)	0.69 (0.4)
brass (cs)	86.2 (1)	54.9 (1)	31.2 (2)	36.4 (1.4)	30.9 (1.4)	76.5 (2.3)	−66.9 (2.5)	20.4 (0.4)	0.66 (0.3)

## Data Availability

The data reported can be shared on demand after contacting the corresponding authors.
